# Violent or competitive? Unpacking adolescent cyber-aggressive behavior in text, video, and game context

**DOI:** 10.3389/fpsyg.2025.1577717

**Published:** 2025-04-25

**Authors:** Yanlei Chen, Lubin Wang, Shaoyang Guo, Qian Gu

**Affiliations:** ^1^School of Educational Science, Liaocheng University, Liaocheng, China; ^2^Psychological Counseling and Therapy Center, Zibo Mental Health Center, Zibo, China; ^3^School of Educational Science, Yangzhou University, Yangzhou, China

**Keywords:** adolescents, violence, competition, cyber-aggressive behavior, social comparison tendency

## Abstract

**Introduction:**

Cyber-aggressive behavior in adolescents is significantly influenced by violence and competition, yet their distinct roles and interactions with contextual and personality factors remain underexplored. This study investigates how violent versus competitive triggers, contextual mediums (text, video, game), and social comparison tendencies shape cyber-aggression.

**Methods:**

Two experimental studies were conducted. Study 1 employed a 2 (violence vs. competition) × 3 (context: text, video, game) mixed design using gamified assessments to measure cyber-aggression. Study 2 expanded this with a 2 (violence vs. competition) × 3 (social comparison tendency: high/medium/low) × 2 (aggression type: verbal/physical) design to dissect behavioral and personality interactions.

**Results:**

Violence consistently elicited higher cyber-aggression than competition across all contexts (Study 1). In Study 2, violent-competitive factors retained significant main effects, while aggression type (verbal/physical) and social comparison tendency alone showed no significant impacts. Key interactions emerged: verbal aggression under competitive conditions provoked stronger cyber-aggression than physical means. High social comparison tendency amplified cyber-aggression in violent contexts, correlating with escalating violence intensity.

**Discussion:**

Violence is the dominant driver of adolescent cyber-aggression, particularly when paired with high social comparison tendencies. Competitive environments, however, disproportionately trigger verbal aggression. These findings highlight the need for context-specific interventions targeting media content and individual predispositions to mitigate cyber-aggressive behavior.

## 1 Introduction

With the deep integration of digitized lifestyle into the lives of adolescents, cyber-aggression has emerged as a significant risk behavior threatening their mental health and social adaptation (Muñoz-Fernández and Sánchez-Jiménez, 2020). According to a 2021 report by the World Health Organization, ~37% of adolescents aged 13–17 globally have experienced cyber-aggression, with 32% of victims exhibiting psychological symptoms such as anxiety and depression (Badura et al., 2024). Unlike traditional aggression, cyber-aggression is characterized by anonymity, contagiousness, and reduced inhibition (Kowalski et al., 2014), and its triggering mechanisms and pathways exhibit unique patterns.

Research has shown that violent elements and competitive elements are two major factors contributing to cyber-aggression among adolescents, but there is no conclusive consensus on which factor plays the predominant role (Gentile et al., 2014). Specifically, individuals often compare their thoughts with others to form self-perceptions. Therefore, when exposed to violent or competitive scenarios, whether individuals exhibit varying levels of cyber aggression due to their distinct social comparison orientations remains a subject of inquiry (Lu et al., 2024). Thus, this research seeks to determine whether violence or competition serves as the primary driver of cyber-aggression by testing distinct violent and competitive contexts, while also exploring how different manifestations of aggression (e.g., physical vs. verbal) influence online behavioral patterns. Additionally, it aims to examine the function of social comparison tendencies in shaping the relationship between exposure to violence/competition and subsequent cyber-aggressive responses.

## 2 Literature review and gaps

### 2.1 Controversy over three theoretical models of cyber-aggression

Cyber-aggression refers to behaviors that use the internet as a medium, utilizing various communication methods and technologies to cause harm to individuals or groups (Zhao and Gao, 2012). This encompasses both aggression triggered by real-world factors and aggression stimulated by engagement in online activities—such as reading web stories, viewing short videos, or excessive gaming—which may escalate hostile behaviors (Oliveira et al., 2024; Tennakoon et al., 2024).

Since the 1980s, researchers have been investigating the psychological mechanisms behind cyber-aggression and proposed three theoretical models. Anderson and Bushman (2002) first systematically explored human cyber-aggressive behavior and proposed the General Aggression Model (GAM), which posits a causal relationship between violence and cyber-aggression, suggesting that exposure to violent content can trigger aggressive behavior. However, the GAM is based on factors related to violent video games, limiting its scope to violent contexts. To extend this theory to non-violent domains, the General Learning Model (GLM) was developed, arguing that cyber-aggressive behavior arises from repeated exposure to violent environments and imitation (Buckley and Anderson, 2006). Both GAM and GLM emphasize external violent factors as primary causes of cyber-aggression. In 2008, Ferguson et al. proposed a different perspective, suggesting that while high-violence environments can induce cyber-aggressive behavior, such behavior persists even when violent elements are removed (Ferguson et al., 2008). They argued that violence serves merely as a direct motivator, with genetic predispositions being the underlying determinant. This led to the development of the Catalyst Model, which posits that individuals inherently prone to violence tend to adopt aggressive patterns, leading to increased cyber-aggression under stress.

These three different theoretical models all suggest that violent factors are the cause of cyber-aggression. However, research findings indicate that while violent video games can increase an individual's tendency toward cyber-aggressive behavior (Anderson and Carnagey, 2009), such games often also contain competitive elements. Therefore, researchers propose that violence is not the sole factor influencing cyber-aggression; competition may also contribute to its occurrence. On this basis, the Competitive Hypothesis was introduced, which argues that violent video games induce cyber-aggressive behavior not because of their violent content but due to the competitive nature inherent in such games. Non-violent games, by contrast, are typically non-competitive. It has been demonstrated in subsequent studies that competitive factors can trigger physiological arousal in individuals, and this arousal leads to aggressive cognitive processes, ultimately resulting in aggressive behavior (Adachi and Willoughby, 2011a,b). In summary, the General Aggression Model (GAM), which is based on violent factors, and the Competitive Hypothesis, which focuses on competitive factors, offer differing explanations for cyber-aggressive behavior. As of now, there is no consensus on whether violence or competition is the primary factor influencing such behavior, indicating further empirical research is needed to resolve this issue.

### 2.2. Violent factor of digital content

Over the past three decades, cyber-aggression has evolved alongside digital technology, progressing from one-way anonymous abuse in the Email Era (e.g., anonymous harassment via emails) to public group confrontations in the Internet Era (Kowalski et al., 2014), where ideological conflicts on platforms fueled “flame wars” and collective attacks (Citron and Norton, 2011). In recent years, its complexity has surged in the Digital Era with technologically sophisticated tactics such as manipulated media, exploitation of software vulnerabilities, and gray industry chains like “paid trolling services”. This trajectory reflects not only escalating technical capabilities but also a deepening societal pervasiveness of hostile online behaviors (Georgakopoulou and Spilioti, 2015).

While digital content manifests in diverse forms—including video games, textual media, and short videos—these platforms converge on shared psychological mechanisms that erode behavioral inhibitions and amplify aggressive tendencies (Nesi et al., 2018). Across these mediums, features such as anonymity-induced moral disengagement, cognitive priming through algorithmic curation, and reward-driven emotional dysregulation collectively weaken users' psychological defenses, creating fertile ground for hostility (Cai, 2016). This vulnerability is particularly evident in violent content consumption: Longitudinal studies demonstrate that repeated exposure to violent games systematically reshapes social cognition, normalizing aggression as both a problem-solving tool and a status-seeking tactic (Anderson and Bushman, 2001; Anderson et al., 2010). While existing research has substantiated the capacity of violent content to elicit aggressive behaviors, it is still unknown which form of carrying violent elements (text, video, game) is more likely to induce aggressive behavior of individuals, and it is worth further research.

### 2.3 Competitive factor of social comparison

Social comparison refers to the process of comparing oneself with others to gain important information about oneself, encompassing both downward and upward comparisons (Jin and Cui, 2013). In digital environments, social comparison is more readily activated. The tendency for social comparison may be a critical factor in triggering cyber aggression. Festinger (1954) proposed that individuals evaluate their abilities and traits through comparisons with others. Digital environments amplify the scope and frequency of these comparisons, as users are more exposed to “idealized” others, thereby intensifying upward social comparison. Upward social comparison can evoke jealousy, frustration, or inferiority, which may lead individuals to alleviate these emotions through aggression. For example, Valkenburg et al. (2006) found that negative social comparisons on social media correlate with adolescent aggression. Fox and Moreland (2015) also demonstrated a significant positive association between social comparison and cyberbullying, with jealousy acting as a mediator. When social comparison threatens self-worth, individuals may resort to demeaning others (e.g., malicious comments) to restore self-esteem (Tandoc et al., 2015). Downward social comparison can also provoke aggression, as individuals attack others perceived as inferior to reinforce their own superiority (Kou et al., 2014). Certain groups in digital environments are more prone to social comparison, exacerbating aggressive behaviors. For instance: Users who deliberately curate a “perfect persona” are more sensitive to comparisons. Individuals with low self-esteem are more likely to exhibit aggression due to social comparison. Platform features like “likes” and rankings further reinforce competitive comparisons.

Social networks serve as a key arena for adolescents to engage in social comparison. Each adolescent constructs a digital identity, and when exposed to others' positive online portrayals, they often compare their own physical or psychological traits to those of “superior” peers (Lei et al., 2023). This process fosters negative self-evaluations (e.g., poor body image) and envy, leading to socially destructive or aggressive behaviors. Moreover, online anonymity and the disinhibition effect reduce behavioral constraints, facilitating aggression. Passive social media use—such as information overload or exposure to “information cocoons”—can also heighten negative emotions (Krasnova et al., 2015). Therefore, this study integrates social comparison into a psychological model of how violence and competition influence aggression, positing that individuals with high vs. low social comparison tendencies exhibit significant differences in aggressive behaviors under violent or competitive conditions.

### 2.4 Literature gaps

This study will address several critical gaps in understanding cyber-aggression through four key research dimensions. First, it seeks to resolve the theoretical debate between the General Aggression Model (GAM) and the Competitive Hypothesis by employing gamified assessments to determine whether violent elements or competitive components better explain mechanisms underlying cyber-aggressive behavior. Second, the research innovatively disentangles violence from competition through distinct experimental stimuli—using Plants vs. Zombies to represent violent contexts and Tetris to exemplify non-violent competition—a methodological improvement over previous studies that conflated these factors. Third, it expands investigation beyond gaming environments to examine how violent and competitive elements manifest differently across varied digital contexts, including textual and video-based online interactions. Fourth, the study introduces novel distinctions by categorizing aggression types (physical vs. verbal) and incorporating social comparison tendencies as a moderating variable. This dual approach will not only clarify how different aggression forms relate to online behavior but also explore how individual differences in social comparison propensity mediate the effects of violent/competitive contexts on cyber-aggression. Through this multifaceted design, the research aims to advance theoretical frameworks while providing practical insights into context-specific prevention strategies.

## 3 Study 1: the influence of violent-competitive factors and context types on cyber-aggressive behavior

### 3.1 Research objectives

Violent and competitive factors are two key elements that trigger cyber-aggression among adolescents; however, no consensus exists regarding which factor is the primary cause. This study aims to further analyze these factors and explore whether violent and competitive factors have different effects on cyber-aggressive behavior across various contexts (textual, video, and gaming scenarios).

### 3.2 Research hypotheses

**H1:** Violent factors play a more significant role in triggering cyber-aggressive behavior compared to competitive factors, which means subjects exposed to violent content demonstrate significantly higher scores in cyber-aggression behaviors than those exposed to competitive content. **H2:** Cyber-aggressive behavior is more likely to occur in video-based contexts, which means subjects exposed to video-based contexts demonstrate significantly higher scores in cyber-aggression behaviors than those exposed to textual-based and game-based contexts.

### 3.3 Research process

#### 3.3.1 Experimental design

A pilot study was conducted with 20 randomly selected participants, none of whom participated in the subsequent formal experiment. Participants were exposed to textual and video materials as well as gameplay trials. Post-exposure interviews confirmed that violent texts, videos, and Plants vs. Zombies successfully elicited violent emotions, while competitive texts, videos, and Tetris effectively induced competitive feelings, ultimately leading to cyber-aggressive behavior.

To exclude the possibility of participants' inherent aggression, violence, and competitive tendencies, three validated scales were employed: The Cyber Aggression Behavior Assessment Scale, the Chinese Adolescent Version of the Buss-Perry Aggression Questionnaire, and the Competitive Attitude Scale. Data from 942 valid adolescent participants were collected to ensure measurement reliability, with the following psychometric properties: (1) The Cyber Aggression Behavior Assessment Scale, developed by Zhao and Gao (2012), originally contains 31 items using a 4-point Likert scale across two subscales (instrumental and reactive aggression). This study utilized the 15-item instrumental aggression subscale, demonstrating excellent reliability (Cronbach's α = 0.967) and satisfactory structural validity indices (χ^2^ = 1473.754, *df* = 187, SRMR = 0.039, CFI = 0.973, TLI = 0.966, RMSEA = 0.090). (2) The Chinese Adolescent Version of the Buss-Perry Aggression Questionnaire, adapted by Lv et al. (2013), comprises 22 items across four subscales (hostility, impulsivity, irritability, and physical aggression) using a 5-point scale (χ^2^ = 3.374, *df* = 2, SRMR = 0.002, CFI = 1.000, TLI = 0.999, RMSEA = 0.075). (3) The Competitive Attitude Scale developed by Chen et al. (2003) consists of 27 items measuring two dimensions (positive competition and excessive competition) on a 5-point scale, with higher scores indicating stronger competitive attitudes (χ^2^ = 8.690, *df* = 2, SRMR = 0.003, CFI = 0.999, TLI = 0.998, RMSEA = 0.103).

The formal experiment adopted a 2 (Violence vs. Competition) × 3 (Context Types: Text, Video, Game) mixed experimental design to study the varies of cyber-aggression. Here, “violence” and “competition” served as within-subject variables, while “context type” acted as a between-subject variable. Participants were randomly divided into two groups using an ABBA sequence to avoid practice effects. Participants are exposed to mindfulness training music between experimental sessions to maintain emotional consistency and minimize external distractions. All participants showed no significant differences in pre-existing aggressive tendencies, violent inclinations, or competitive attitudes.

The Spiciness Level in the Hot Sauce Paradigm (Lieberman et al., 1999) was used to measure cyber-aggressive behaviors. In psychological research, the Hot Sauce Paradigm is a classic laboratory (Lieberman et al., 1999) method for measuring aggressive behavior. It quantifies aggression by asking participants to allocate hot sauce to others (knowing they dislike spicy food). Recent studies have adapted this paradigm to assess cyber-aggression (Gao et al., 2021; Quan et al., 2024). The specific scenario in the Hot Sauce Questionnaire is described as follows: “If you had the opportunity to add hot sauce to someone else's meal via online instructions, with seven spiciness levels (1–7) gradually increasing in intensity, what level would you choose?” Participants responded via mobile devices using a 7-point scale, where higher scores indicated greater levels of cyber aggression.

#### 3.3.2 Experimental participants

Using G^*^Power 3.1.9.7 software, the required sample size was calculated with $\eta_{p}^{2}$ = 0.25, resulting in a target of 86 participants to achieve a statistical power of 0.80. A total of 90 adolescents from 942 valid samples in pretests were recruited, with all 90 datasets being valid. All participants were voluntary recruits who met the following criteria: physical and mental health with no history of psychological disorders, normal vision, no dyslexia, no specific gaming preferences, and no prior participation in similar psychological experiments. After the experiment, participants were debriefed on the research objectives, with an emphasis that “aggressive behavior” was solely a measured variable and that all task scenarios were fictional, aiming to alleviate psychological concerns. To ensure post-experiment wellbeing, participants received positive psychological counseling and were provided with the research team's contact information and access to a free psychological counseling hotline, available for assistance within one week of participation.

#### 3.3.3 Experimental materials

The materials of textual, video, and gaming contexts are listed in Appendix. The experimental contexts were categorized into three types: (1) Textual Context, including violence-oriented material (a written scenario of a daughter developing depression due to physical abuse by her father) and competition-oriented material (a written scenario of a daughter becoming depressed after being belittled by her father through unfavorable comparisons with others); (2) Video Context, featuring violence-oriented material (a video clip showing a mother physically assaulting her daughter) and competition-oriented material (a video clip depicting a mother using social comparison tactics to demean her child); and (3) Gaming Context, comprising a violence-oriented game (Plants vs. Zombies, where players strategize to defend their home by defeating invading zombies) and a competition-oriented game (Tetris multiplayer mode, a timed challenge where players compete to outscore opponents).

#### 3.3.4 Experimental procedures

Participants were randomly assigned to Group A and Group B and allocated to separate classrooms for experimental procedures involving three distinct contexts (text, video, and game). All materials were delivered digitally through computer interfaces, with Group A exposed to violent text narratives while Group B received competitive text materials in the textual context. Subsequently, Group A watched violent video content with headphones while Group B viewed competitive video content during the video context phase. For the gaming context, Group A played the violent game Plants vs. Zombies while Group B engaged in the multiplayer competitive game Tetris. Each 20-min exposure phase was immediately followed by online completion of the hot sauce questionnaire. After completing all three contexts, participants underwent emotional regulation through soothing music before experiencing reversed material exposure: Group A interacted with competitive materials while Group B received violent counterparts for another 20-min session, culminating in a final online questionnaire submission.

#### 3.3.5 Research results

The descriptive statistics of Study 1 are presented in Table 1.

**Table 1:** The mean and corresponding standard deviation for Study 1.

**Variables (*N* = 90)**	**Hot sauce scores**	
	**Violence**	**Competition**
Textual (*N* = 30)	3.47 (2.34)	2.43 (2.16)
Video (*N* = 30)	4.87 (2.09)	3.50 (2.01)
Gaming (*N* = 30)	3.13 (2.08)	2.63 (1.71)

To further check the results, a mixed experimental design of 2 (Violence vs. Competition) × 3 (Context Types: Textual, Video, Gaming Scenarios) was analyzed using repeated measures analysis of variance. The Mauchly's test for sphericity yielded significant results, indicating that the assumption of sphericity was violated. Therefore, the Greenhouse-Geisser correction was applied to adjust the degrees of freedom. The results in Table 2 indicated a significant main effect of Violence-Competition [*F*_(1, 42)_ = 33.963, *p* < 0.001, $\eta_{p}^{2}$ = 0.281], which means subjects exposed to violent content demonstrate significantly higher scores in cyber-aggression behaviors than those exposed to competitive content (*M*_violence_ = 3.82, *M*_competition_ = 2.85, mean difference = 0.97, *p* < 0.001). Thus, the Hypotheses H1 has been supported. Furthermore, the main effect of Context Type was also significant [*F*_(2, 32)_ = 4.354, *p* < 0.05, $\eta_{p}^{2}$ = 0.091], which means subjects exposed to video-based contexts demonstrate significantly higher scores (*M*_video_ = 4.19, *M*_text_ = 2.95, *M*_Gaming_ = 2.88) in cyber-aggression behaviors than those exposed to textual-based (mean difference = 1.24, *p* < 0.05) and game-based contexts (mean difference = 1.31, *p* < 0.05). Thus, the Hypotheses H2 has been supported. Additionally, the interaction between Violence-Competition and Context Type was not significant.

**Table 2:** Two-factor mixed design ANOVA results for Study 1.

**Source**	***SS***	***df***	***MS***	***F***	*** $\eta_{p}^{2}$ ***
1. Between-subjects effect	2,713.5	90			
2. Within-subjects effect	155.5	90			
3. A (context type)	64.311	2	32.156	4.354^*^	0.091
4. B (violence vs. competition)	42.050	1	42.050	33.963^***^	0.281
5. A × B	5.733	2	2.867	2.315	0.051
6. Total	2,869	180			

N = 90.

^*^p < 0.05.

^***^p < 0.001.

### 3.4 Brief discussion

From the analysis of data in this experiment, it can be seen that the main effect of “Violence vs. Competition” is significant. In different types of contexts, the level of cyber-aggressive behavior triggered by violent factors is significantly higher than that induced by competitive factors. This aligns with our hypothesis and previous research findings, further supporting the General Aggression Model, which posits a causal relationship between violent factors and cyber-aggressive behavior (Anderson and Bushman, 2002).

From the main effects graph and *post hoc* test results, it is evident that the level of cyber-aggressive behavior in video contexts is significantly higher than in textual and gaming contexts. Compared to textual contexts, video contexts provide more emotional cues (such as background music and actors' performances), making it easier for participants to quickly arouse their own emotional experiences and induce higher levels of cyber-aggressive behavior. Compared to video contexts, gaming contexts require participants to actively engage in games and impose punishments on game opponents; however, when imposing punishments, participants are influenced by the social approval effect. This result may be understood from the following perspectives: aggressive behavior, as a negative act that exceeds societal norms, is not socially supported or accepted. People's behaviors and beliefs inherently seek social conformity, leading participants to conceal their aggressive behaviors, thus resulting in lower levels of aggression in gaming contexts compared to video contexts.

Study 1 explored whether violent factors or competitive factors triggered cyber-aggressive behavior and the impact of violent vs. competitive factors on cyber-aggressive behavior across different contexts. The research results indicate that violent factors are the decisive factor in triggering cyber-aggressive behavior. Aggression can be categorized into two types: verbal aggression and physical aggression. Therefore, it is important to investigate whether different forms of aggression have varying effects on individuals' cyber-aggressive behavior. Additionally, the study found that individuals often compare their thoughts with those of others to gain self-knowledge, implying that when exposed to violent or competitive situations, individuals with different social comparison tendencies may exhibit varying levels of cyber-aggressive behavior. Consequently, in subsequent experiments, the variable “social comparison tendency” will be introduced. Therefore, Study 2 will further investigate the effects of violent vs. competitive factors, types of aggression, and social comparison tendencies on cyber-aggressive behavior.

## 4 Study 2: the impact of violent-competitive factors, social comparison tendencies, and aggression types on cyber-aggressive behavior in textual contexts

### 4.1 Research objectives

The results from Study 1 indicated that violent factors are the primary determinants of cyber-aggressive behavior, while online aggression can be categorized into two types: verbal aggression and physical aggression. Therefore, Study 2 aims to explore whether different forms of aggression influence individuals' levels of cyber-aggressive behavior. Additionally, since individuals often compare their thoughts with those of others to gain self-knowledge, this study investigates whether individuals exhibit varying levels of cyber-aggressive behavior due to differences in their social comparison tendencies when exposed to violent or competitive situations.

### 4.2 Research hypotheses

**H3:** Under competitive conditions, verbal aggression induces greater cyber-aggression than physical aggression, with subjects exposed to verbal aggression exhibiting significantly higher cyber-aggression scores than those exposed to physical aggression. **H4:** Under violent conditions, cyber-aggression levels are influenced by social comparison tendency, as subjects with high social comparison tendency demonstrate significantly higher cyber-aggression behaviors than their low social comparison counterparts.

### 4.3 Research process

#### 4.3.1 Experimental design

A mixed experimental design was employed: 2 (Violence vs. Competition) × 3 (Social Comparison Tendency: High, Medium, Low) × 2 (Aggression Type: Verbal Aggression, Physical Aggression). Here, “Violence vs. Competition” and “Aggression Type” were within-subjects variables, while “Social Comparison Tendency” was a between-subjects variable. The Spiciness Level in the Hot Sauce Paradigm was used to measure cyber-aggressive behaviors (see Section 3.3.1). Moreover, participants were categorized into three groups based on their social comparison tendencies: high, medium, and low. Grouping was determined by dividing participants according to the upper 27% (high), medium 46%, and lower 27% (low) of the social comparison tendency scores. Specifically, Study 2 used the social comparison tendency scale developed by Wang et al. (2006) with seven 5-point Likert items to measure the level of social comparison tendency (Cronbach's α = 0.88, CFI = 0.96, GFI = 0.95, AGFI = 0.96, RMSEA = 0.05). To minimize practice effects, an ABBA sequence was used, and participants were randomly assigned into two groups for the formal experiment. Music was prepared to help soothe participants' emotions between experimental sessions, ensuring that their emotional states remained consistent and were not influenced by external factors.

#### 4.3.2 Experimental participants and materials

Using G^*^Power 3.1.9.7 software, the required sample size was estimated with an effect size ($\eta_{p}^{2}$) of 0.25, indicating that 28 participants would achieve a statistical power of 0.80. In this experiment, 32 undergraduate students from 942 valid samples in pretests were randomly recruited and participated in the study. The other ethical requirements of the subjects were consistent with the design of Experiment 1. In addition, the textual material was used to evoke cyber-aggressive behaviors, including violence-oriented material (a written scenario of a daughter developing depression due to physical abuse by her father) and competition-oriented material (a written scenario of a daughter becoming depressed after being belittled by her father through unfavorable comparisons with others).

#### 4.3.3 Experimental procedures

Participants were randomly divided into two groups, Group A and Group B, and placed in separate classrooms. First, participants were shown a projection of the QR code of the questionnaire for social comparison tendency, allowing them 5 min to complete it using their smartphones. Next, Group A received the violent text material online, while Group B received the competitive text material online. Participants were given 10 min to read and answer the questions following the materials. After completing these tasks, the questionnaires were collected. Soothing music was played to help participants relax and return to a normal emotional state. Once their emotions stabilized, Group A received the competitive text material online, and Group B received the violent text material online. Participants then spent another 10 min reading and answering questions based on the materials provided. After completing this second task, the questionnaires were collected. Finally, after the experiment concluded, participants were briefed on the purpose of the study, thanked for their participation, and given a small gift as a token of appreciation.

### 4.4 Research results

The descriptive statistics of Study 2 are presented in Table 3.

**Table 3:** The mean and corresponding standard deviation for Study 2.

**Variables (*N* = 32)**	**Groups of social comparison tendency**		
	**Low**	**Medium**	**High**
Violence verbal aggression	5.80 (3.39)	7.00 (2.62)	8.30 (1.05)
Violence physical aggression	5.20 (3.58)	7.50 (1.67)	8.40 (1.35)
Competition verbal aggression	5.10 (3.41)	5.58 (2.53)	5.70 (2.49)
Competition physical aggression	4.30 (3.16)	5.25 (1.91)	4.00 (2.05)

To further check the results, a mixed ANOVA with a 2 (Violence vs. Competition) × 3 (Social Comparison Tendency: High, Medium, Low) × 2 (Aggression Type: Verbal Aggression, Physical Aggression) design was conducted to analyze the data. Mauchly's test of sphericity was significant; therefore, the results from within-subjects tests in Table 4 were referenced. The analysis revealed a significant main effect of Violence vs. Competition [*F*_(1, 29)_ = 52.196, *p* < 0.001, $\eta_{p}^{2}$ = 0.643], which means subjects exposed to verbal aggression exhibiting significantly higher cyber-aggression scores than those exposed to physical aggression (*M*_violence_ = 7.03 vs. *M*_competition_ = 4.99, mean difference = 2.04, *p* < 0.001). Neither Aggression Type [*F*_(1, 7)_ = 1.689, *p* = 0.204] nor Social Comparison Tendency [*F*_(2, 26)_ = 51.413, *p* = 0.26] showed significant main effects.

**Table 4:** Two-factor mixed design ANOVA results for Study 2.

**Source**	***SS***	***df***	***MS***	***F***	*** $\eta_{p}^{2}$ ***
1. Between-subjects effect	5,176.95	32			
2. Within-subjects effect	411.748	96			
3. A (social comparison tendency)	52.088	2	26.044	1.413	0.089
4. B (violence vs. competition)	132.769	1	132.769	52.196^***^	0.643
5. C (verbal vs. physical)	7.083	1	7.083	1.689	0.055
6. A × B	37.202	2	18.601	7.313^**^	0.335
7. A × C	5.258	2	2.629	0.627	0.041
8. B × C	7.083	1	7.083	8.661	0.23
9. A × B × C	3.252	2	1.626	1.988	0.121
10.Total	5,588.719	128			

*N* = 90.

^**^p < 0.01.

^***^p < 0.001.

**Figure 1:**
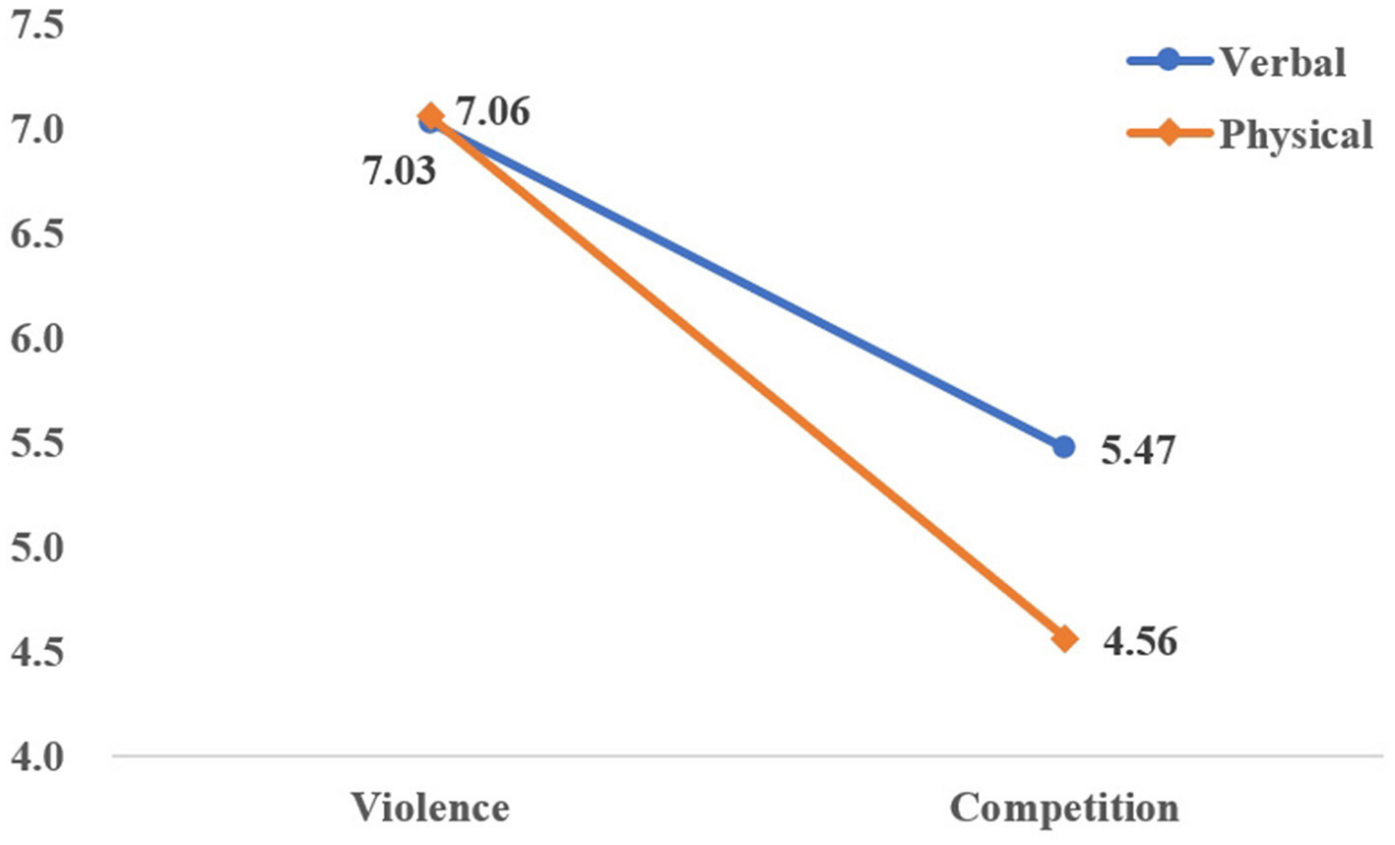
Interaction of violence-competition and aggression types.

**Figure 2:**
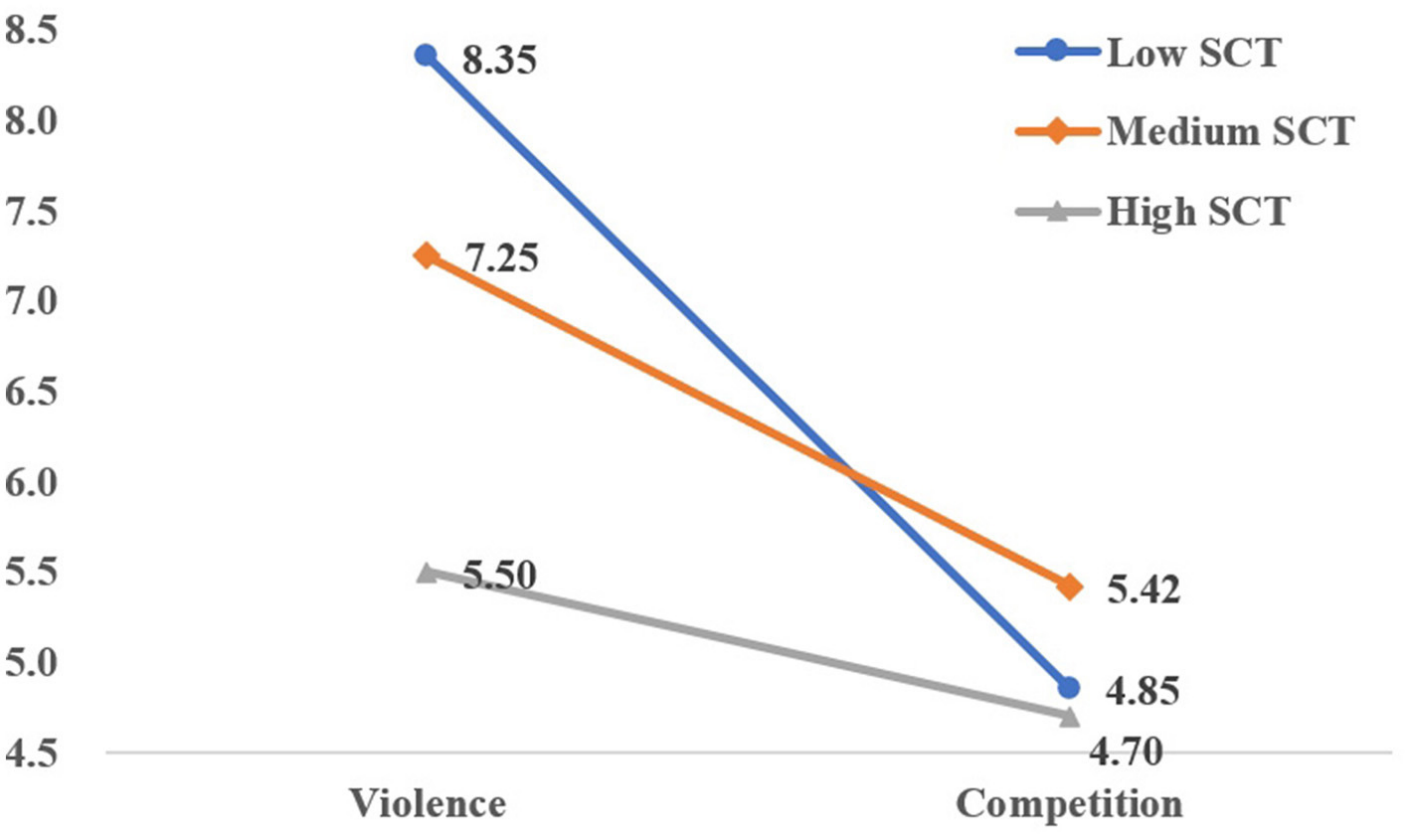
Interaction of violence-competition and social comparison tendency.

Notably, significant two-way interactions emerged between Violence vs. Competition and Aggression Type [*F*_(1, 7)_ = 8.661, *p* < 0.01, $\eta_{p}^{2}$ = 0.230; Figure 1], as well as between Violence vs. Competition and Social Comparison Tendency [*F*_(2, 18)_ = 7.313, *p* < 0.01, $\eta_{p}^{2}$ = 0.335; Figure 2]. However, the three-way interaction among Violence vs. Competition, Social Comparison Tendency, and Aggression Type failed to reach significance [*F*_(2, 1)_ = 1.988, *p* = 0.155, $\eta_{p}^{2}$ = 0.121; Table 3].

Due to the significant interaction effects, further simple effects analyses of cyber-aggressive scores were conducted to examine the specific patterns. The significant interaction between Violence vs. Competition and Aggression Type was analyzed: Under competitive conditions, the cyber-aggressive scores of verbal aggression were significantly higher than those from physical aggression (Mean difference = 0.944, *p* < 0.05), indicating that Hypotheses H3 has been supported. Under violent conditions, no significant difference was found in selection rates between verbal and physical aggression (Mean difference = 0.00, *p* = 1.00 > 0.05).

The significant interaction between Violence vs. Competition and Social Comparison Tendency was analyzed: Under violent conditions, the cyber-aggressive scores of high social comparison tendency were significantly higher than those from the group of low social comparison tendency (Mean difference =2.850, *p* < 0.05), indicating the Hypotheses H4 has been supported. Moreover, no significant differences were found between other groups: (1) Low vs. medium: Mean difference = −1.75, *p* = 0.225 > 0.05; (2) Medium vs. high: Mean difference = −1.10, *p* = 0.605 > 0.05. Under competitive conditions, no significant differences were found among groups: (1) Low vs. Medium: Mean difference = −0.717, *p* = 0. 855 > 0.05; (2) Low vs. high: Mean difference = −0.150, *p* = 0.999 > 0.05; (3) Medium vs. high: Mean difference = 0.567, *p* = 0.990 > 0.05.

### 4.5 Brief discussion 2

The results of the data analysis indicate that, under competitive conditions, verbal aggression is more likely to provoke cyber-aggressive behavior compared to physical aggression. These effects can be attributed to the following reasons:

Ubiquity of verbal aggression: As legal awareness becomes increasingly ingrained in society, most individuals understand that physical aggression is explicitly prohibited by law. Victims of such aggression are also more likely to use legal means to protect themselves. Consequently, overt forms of physical aggression (e.g., physical assault or restraint) have become less common. On the other hand, verbal aggression, which lacks an obvious direct harm dimension, is harder to regulate and adjudicate under the law. As a result, verbal violence has become increasingly prevalent in society (Li et al., 2007).

Severity of consequences for verbal aggression: Unlike physical aggression, verbal aggression inflicts long-term psychological harm by tormenting victims mentally and emotionally. This type of aggressive behavior is particularly insidious because it often goes unnoticed or unaddressed due to its covert nature. Over time, verbal attacks—such as insults, curses, or mocking remarks—can damage interpersonal trust, negatively impact emotional wellbeing, and even lead to long-term psychological issues like anxiety and depression (Wang et al., 2014).

In the context of violent conditions, individuals with high social comparison tendencies exhibit higher levels of cyber-aggressive behavior. This can be explained by the following reasoning: high social comparison tendency individuals often use social comparison as a means to enhance their self-evaluation and satisfaction. These individuals are motivated to engage in frequent social comparisons to maintain their self-esteem (Jin et al., 2017). In violent environments, such individuals perceive higher levels of threat compared to those with low social comparison tendencies. To defend and preserve their self-esteem, they are more likely to exhibit high levels of aggressive behavior as a form of self-compensation.

The second experiment explored the influence of violence vs. competition factors, aggression type, and social comparison tendency on cyber-aggressive behavior. The results demonstrated an interaction between violent vs. competitive factors and social comparison tendency: under violent conditions, individuals with high social comparison tendencies exhibited higher levels of cyber-aggressive behavior. Furthermore, previous studies have shown that exposure to violent environments does not inevitably lead to cyber-aggressive behavior; individual perceptions of discrimination play a critical role in shaping such behaviors (Fu et al., 2016). Therefore, future research should explore how individuals' perceptions of discrimination influence the relationship between violent vs. competitive factors and cyber-aggressive behavior.

Building on these findings, Study One and Two have demonstrated through gamified assessments that violence is a primary factor in provoking cyber-aggressive behavior. Additionally, interactions between violent vs. competition factors and social comparison tendency were identified. To further validate these experimental results, the third study will adopt a measurement-based approach to explore the combined effects of violence, competition, social comparison tendency, and perceptions of discrimination on cyber-aggressive behavior. Furthermore, it aims to establish a theoretical model that integrates these variables into a cohesive framework for better understanding their interrelationships.

## 5 Overall discussion

### 5.1 The impact of violent and competitive factors on cyber-aggressive behavior

In Study 1, a gamified assessment method was employed to examine the effects of violent and competitive factors on cyber-aggressive behavior across different contexts (textual, video, and gaming). The results revealed that regardless of the context, violent factors were the primary determinants of cyber-aggressive behavior. These findings align with those of Engelhardt et al. (2011) and are consistent with predictions from the General Aggression Model (GAM), which posits that aggressive behaviors arise from learning, activating, and applying knowledge structures related to aggression stored in memory.

When individuals are exposed to violent contexts, their inherent traits interact with the violent factors, influencing their internal states. After evaluating these states, individuals may engage in aggressive behavior. Long-term reinforcement of aggressive prevention can not only make individuals more prone to aggression but also create a cognitive schema linking violence and aggression, ultimately shaping an aggressive personality. Specifically, when individuals encounter violent situations online (e.g., being insulted in chat or killed in a game), their physiological systems are aroused (e.g., increased heart rate, dry mouth). The individual internally perceives the violent situation as hostile and evaluates it, leading to a hostile internal state (e.g., anger). This ultimately prompts the individual to retaliate online, thereby inducing cyber-aggressive behavior.

Violent factors induce cyber-aggressive behavior, and prolonged or repeated exposure to violent online environments reinforces this tendency. Through such reinforcement, individuals learn to counteract violence, develop cognitive scripts and schemas related to aggression, and automatically activate aggressive schemas in subsequent encounters with online violence, resulting in cyber-aggressive behavior.

### 5.2 The role of social comparison tendency

Study 2 investigated the influence of social comparison tendencies on violent factors and cyber-aggressive behavior, revealing an interactive effect between social comparison tendencies and violence. Under violent conditions, the level of cyber-aggression was influenced by social comparison tendencies, with higher levels of social comparison leading to greater cyber-aggressive behavior compared to lower levels.

Individuals have a fundamental need to actively maintain self-evaluations, often engaging in social comparisons to satisfy their need for self-esteem maintenance. Compared to those with low social comparison tendencies, individuals with high social comparison tendencies frequently engage in social comparisons in daily activities such as learning and work, using it as a means to enhance self-evaluation and satisfaction (Heatherton and Wyland, 2003). Self-compensation theory suggests that when external environments pose threats, individuals tend to adopt measures to compensate for their shortcomings (Jin et al., 2017).

In online violent contexts, individuals may feel anxious due to fear. Compared to those with low social comparison tendencies, individuals with high social comparison tendencies experience stronger anxiety and threat in violent situations, accompanied by negative reactions such as decreased self-efficacy and reduced self-esteem (Liu, 2021). The discrepancy between an individual's real self and ideal self creates psychological gaps, prompting individuals to compensate for these deficits. To maintain self-evaluation and restore self-esteem, high social comparison individuals adopt compensatory strategies, engaging in cyber-aggressive behavior to address threats, regain a sense of self-worth, and preserve or enhance their self-evaluation (Gong and Zhang, 2020).

### 5.3 Future intervention strategies

Controlling cyber-aggressive behaviors in digital environments requires a multidimensional governance framework integrating technological interception, legal deterrence, educational guidance, and psychological support. Technologically, platforms should establish violence detection systems through natural language processing (NLP) to analyze emotional valence and aggressive lexicon, enabling real-time detection of high-risk behaviors such as verbal abuse and doxxing. Machine learning models could identify inflammatory “bandwagon” rhetoric (Bilewicz et al., 2021; Sportelli et al., 2025), automatically collapsing contentious comments or labeling disputed content. Implementing account credit scoring systems would restrict privileges for users repeatedly posting aggressive content, while features like “stranger message blocking” and tiered comment permissions empower users to manage interactions. Mandatory “safe mode” for adolescent accounts should filter violent content, coupled with prioritized reporting mechanisms and incentives for creating prosocial content (Stoilova and Livingstone, 2021). Legally, platforms must enforce real-name verification and pre-moderation of violence-related posts, permanently banning malicious rumor-mongers and prosecuting severe cases. Cross-border collaboration through Interpol could enhance global accountability. Educationally, integrating digital literacy curricula with case-based simulations in schools cultivates critical thinking to distinguish legitimate critique from cyber violence. Finland's KiVa anti-bullying program (Salmivalli et al., 2011) demonstrates effectiveness through school-wide interventions and virtual role-playing, reducing cyberbullying by 50%. The findings from a reinforcement learning model analyzing a large dataset of Instagram usage records reveal that adolescents exhibit higher sensitivity to social feedback compared to adults, demonstrating greater responsiveness to social media engagements like “likes,” which directly influences their online engagement levels and emotional states (da Silva Pinho et al., 2024). Comprehensive support systems should include AI counseling hotlines and trauma-informed legal aid, while implementing digital behavioral correction for juvenile offenders through consequence documentaries and anti-bullying community service (Hangartner et al., 2021).

## Data Availability

The raw data supporting the conclusions of this article will be made available by the authors, without undue reservation.
