# Supplementary material for: Violent or competitive? Unpacking adolescent cyber-aggressive behavior in text, video, and game context
Source: Front Psychol. 2025 Apr 25;16:1577717. doi: 10.3389/fpsyg.2025.1577717 (PMC12061860; doi:10.3389/fpsyg.2025.1577717)

Supplementary Material

This appendix lists the materials of textual, video, and gaming context.

# Textual Context

The textual context is from consulting records of virtual character Wang Jie.

## Violent Text

**Counselor:** Welcome to the counseling session. What aspects do you need our help with this time?

**Wang Jie:** I...want to die.

**Counselor:** Can you tell me what made you have such thoughts?

**Wang Jie: (**long silence) I failed the college entrance exam, and my mom beat me up badly. Ever since I was little, that woman has always been unhappy. Whenever she's unhappy, I'm the one who suffers. It's not that I'm disobedient; I'm very obedient. Whenever someone asks about my injuries, I always tell them I fell by accident.

But even though I'm so obedient, that woman still doesn't like me. She always calls me stupid, an idiot, and a good-for-nothing. I'm not allowed to like singing or extracurricular books; I can only like English words, Euler's Law, and equations. I don't have any chance to relax. I feel like a clock hanging on the wall, constantly ticking without stopping, not even for a second!

Whenever I slack off a little, all I get is a beating. Now, whenever I hear her call my name in a raised tone, I shake with fear. At first, I hated myself, wondering why I didn't do well on the exam this time, why I didn't try harder, why I couldn't make her happy.

But now I understand that it's her I should hate! That woman! She doesn't love me at all! What do I have to do to make her love me? A score of 100? A key high school? A prestigious university? Am I unworthy of her love just because I can't get a perfect score? Am I not her daughter anymore just because I can't get into a prestigious university?

Does she know how hard I've tried? How desperate I am? I've had enough! Since no matter what I do, all I get is beatings, I give up. I might as well be useless; I might as well die. I just want to tell her, Mom, I don't want to be your daughter anymore!

...

**Diagnosis:** Due to severe psychological trauma caused by her mother's violent behavior, she has been diagnosed with major depressive disorder.

**If you have the chance to add chili sauce to her father's food, with chili sauce ranging from levels 1 to 7 in increasing spiciness, and knowing that the person cannot handle spicy food, would you:**

**(1) Add a spiciness level of 1 2 3 4 5 6 7 to an unrelated stranger**

**(2) Add a spiciness level of 1 2 3 4 5 6 7 to the mother in the text**

## Competitive Text

**Counselor:** Welcome to the counseling session. What aspects do you need our help with this time?

**Wang Jie:** I...want to die.

**Counselor:** Can you tell me what made you have such thoughts?

**Wang Jie:** (silent for a long time) I failed the college entrance exam, and my mom said I couldn't compare to her colleagues' kids, that I embarrassed her. Ever since I was little, that woman has always been like this, comparing me to others in everything - academics, talents, anything that could be compared.

And maybe I never won in her comparisons, so she always said I was a kid who didn't bring her honor. Back then, to satisfy her vanity for comparison, I started endless studying. I didn't dare to stop, to get tired, or to fall behind. All of this was just to gain her approval, even if it was just an approving glance.

But I was wrong. All I got from her was praise for other people's kids and criticism for my own. Why? Why could she trample on my self-esteem like this? Why could she ignore my efforts? Why could she be so stingy with her praise? I felt like I was sinking to the bottom of the water, my limbs flailing helplessly, my heart aching and battered, struggling to breathe.

I understand that I will never be the kind of kid who brings her honor. I will always live under that woman's comparisons and criticism. I will always bear pain that I can't handle. This is a hopeless way that I can see to its end. So, I give up. I might as well be useless. I might as well die. I just want to tell her, Mom, I don't want to be your daughter anymore.

...

**Diagnosis:** Due to severe psychological trauma caused by the mother's behavior of suppressing the child through comparisons with others, a diagnosis of major depressive disorder has been confirmed.

**If you have the chance to add chili sauce to her father's food, with chili sauce ranging from levels 1 to 7 in increasing spiciness, and knowing that the person cannot handle spicy food, would you:**

**(1) Add a spiciness level of ____ to an unrelated stranger (choose from 1, 2, 3, 4, 5, 6, 7)**

**(2) Add a spiciness level of ____ to the mother in the text (choose from 1, 2, 3, 4, 5, 6, 7)**

# Video Context

## Violent Outlines

The video is excerpted from the Japanese drama "Being Good"(きみはいい子) published by Arc Entertainment. Here is an outline of the video:

**Girl (on the phone with Dad):** No! No! I don’t wanna!

**Mom:** Ayane, stop acting spoiled with Dad.

**Girl:** I want Daddy to come home! Daddy, come home now!

**Mom (snatches the phone):** Hey, you promised you’d come back this month.

**Girl (crying in the background):** Why won’t Daddy come home?

**Mom:** Leave those things to the people on-site.

**Girl (screaming):** Daddy! Daddy! Daddy!

**Mom:** Hmm, I get it.

**Girl:** No! Why won’t he come back?!

**Mom:** I’ll talk to Ayane. Yeah, goodbye.

**Girl:** No! Noooo!

**Mom (pushes the girl down): S**o annoying. Shut up.

(The girl starts crying.)

**Mom (throws crayons at her):** Shut up! Stop crying! You’re so annoying!

(Later, the girl spills milk at the dining table and glances nervously at her mom.)

**Mom (turns off the TV, slaps the girl, and throws a rag on the floor):** Clean it up.

(The girl climbs down and starts wiping the floor.)

(The girl stands frozen at the entrance after returning home. The mom yanks off her shoes, drags her by the collar into the house, and slaps her.)

**Girl (covering her head, whimpering):** I’m sorry… I’m sorry…

**Mom (yelling, throws a remote at her):** Shut up! It’s because you’re always causing trouble!

(The girl runs to her room crying. The mother chases her, pins her to the floor, and beats her.)

**Mom (hitting her):** Stay still! Understand? Why do you do these things? Useless nonsense! Hear me? Why don’t I do things like this?!

**Girl (sobbing):** I’m sorry… I’m sorry…

**Mom (continues hitting):** Do you get it?! Do you know why I’m hitting you?! Why apologize? What good is that?! Explain yourself properly! Don’t embarrass me! Why… why do you keep causing trouble?!

(The girl curls up and stops crying. The mom lowers her fists.)

**Mom:** Stand up! Stand up!

(When the girl doesn’t move, the mom yanks her up and forces her to look at her.)

**Mom:** I said stand up! What happens when you misbehave? Now you know.

(The mom raises her hand to hit again. The girl shields her head. The mom slowly lowers her arm and leaves the room, abandoning the girl.)

## Competitive Outlines

The video is excerpted from the Chinese drama "A Little Dilemma"(小舍得) directed by Xiaobo Zhang. Here is an outline of the video:

**Mi Tao:** Huanhuan, let’s team up.

**Huanhuan:** Manman, let’s play together!

**P.E. Teacher:** Don’t swing the rope near others!

**P.E. Teacher:** Focus on the key points I mentioned earlier—rhythm!

**Students:** Four, five, six, seven!

(Next scene)

**Mi Tao:** Mom, Huanhuan is bullying me at school.

(Next scene)

**Huanhuan’s Mom:** Explain yourself. Why did you treat Mi Tao that way?

**Huanhuan: I** didn’t do anything.

**Huanhuan’s Mom: T**eacher Zhang already called and told us everything.

**Huanhuan’s Dad:** We want to understand why, sweetheart.

**Huanhuan:** I’m just… I’m just upset!

(Crying) Why do you always praise her? No matter what I do, it’s never as good as her.

Even when I get first place, her first place is better!

**Huanhuan’s Mom:** If a classmate is better than you, you should work harder and learn from them.

Strive to match their level. How could you bully her? Clearly, the teacher wasn’t wrong about this.

**Huanhuan’s Dad:** Tomorrow at school, you must apologize to Mi Tao.

**Huanhuan’s Mom:** Not tomorrow—right now. Go now. Apologizing immediately shows sincerity.

**Huanhuan’s Dad:** It’s late! What time do you think it is?

**Huanhuan:** No! No! Why should I apologize to her? I didn’t do anything wrong! Who I’m friends with is none of your business! I just don’t want to be friends with her, okay?

**Huanhuan’s Mom:** You…!

**Huanhuan:** The teacher said well-rounded qualities are what truly matter. But you only praise her and compare me to her all the time. I hate you!

**Huanhuan’s Mom:** Don’t think yelling makes you right. This ends here, understand? You refuse to admit you’re wrong? Fine, I’ll confiscate all your dolls. You’ll get them back when you apologize.

**Huanhuan:** Take them then! Why don’t you throw me away too?! Go adopt Mi Tao—she’s better at everything anyway!

**Huanhuan’s Mom:** Oh, you’re so clever, aren’t you?

**Huanhuan:** (Chasing her mom) Mom, don’t! Don’t throw them! Please!

**Huanhuan’s Mom:** Let go of me! Get away!

**Huanhuan’s Dad:** Lili, scold her if you must, but don’t throw her dolls!

**Huanhuan’s Mom:** Stay out of this! Shut up, or she’ll never learn!

**Huanhuan:** No! No!

**Huanhuan’s Mom:** Let. Go.

**Huanhuan:** Mom, I’m sorry! Don’t take my dolls!

**Huanhuan’s Mom:** Apologizing to me means nothing. You hurt Mi Tao.

**Huanhuan:** Fine! I’ll apologize to Mi Tao! Just don’t take my dolls!

(Next scene)

**Huanhuan’s Dad:** We’re here today to apologize to Mi Tao. Mi Tao, we’re sorry. Uncle promises this will never happen again, okay?

**Mi Tao:** I was wrong too. I shouldn’t have pushed you. Huanhuan, I’m sorry.

**Huanhuan’s Mom:** (To Huanhuan) What did we agree on at home? What are you supposed to do?

**Huanhuan’s Dad:** Give her time. She needs to gather her thoughts. (To Mi Tao) Sorry.

**Mi Tao’s Mom:** It’s fine, really.

**Huanhuan’s Mom:** Huanhuan.

**Huanhuan:** Mi Tao… I’m sorry. I apologize.

**Mi Tao’s Mom:** It’s okay, Huanhuan. Don’t worry.

**Huanhuan’s Mom:** Don’t coddle her. This is a matter of principle. She needs to understand how serious this is. (To Huanhuan) And?

**Huanhuan:** You… you study better than me. You’re smarter. I should learn from you and try to be like you… not be jealous.

**Huanhuan’s Mom:** (Coldly) "I hope you can forgive me."

**Huanhuan:** (Mumbling) I… hope you can forgive me. Will you forgive me?

**Mi Tao:** It’s okay, Huanhuan. I forgive you.

**Huanhuan’s Mom:** Mi Tao is so mature.

**Mi Tao’s Mom:** It’s nothing, really.

**Huanhuan’s Dad: I**t’s settled now.

**Huanhuan’s Mom:** (Sighs) Huanhuan, you…

**Huanhuan’s Dad:** Sorry again.

**Mi Tao’s Mom:** Go check on her.

**Huanhuan’s Mom:** Our apologies.

**Mi Tao’s Mom:** No need.

(Next scene)

**Huanhuan’s Dad:** Huanhuan! Huanhuan!

**Huanhuan’s Mom:** What are you running for? Think you’re in the right? Did you see Mi Tao’s living conditions? And yours? She isn’t jealous of you.

**Huanhuan:** I said everything you made me say! What else do you want?!

**Huanhuan’s Mom:** Have you even reflected on yourself? We’ve given you everything—food, clothes, a good life—no worries at all. Where’s your focus? Is it on your studies? No! You’re too busy competing with others. So you bully them instead?

**Huanhuan’s Dad:** Huanhuan…

## Gaming Context:

**Violent Game: Plants vs. Zombies.**

**Game screenshot:**


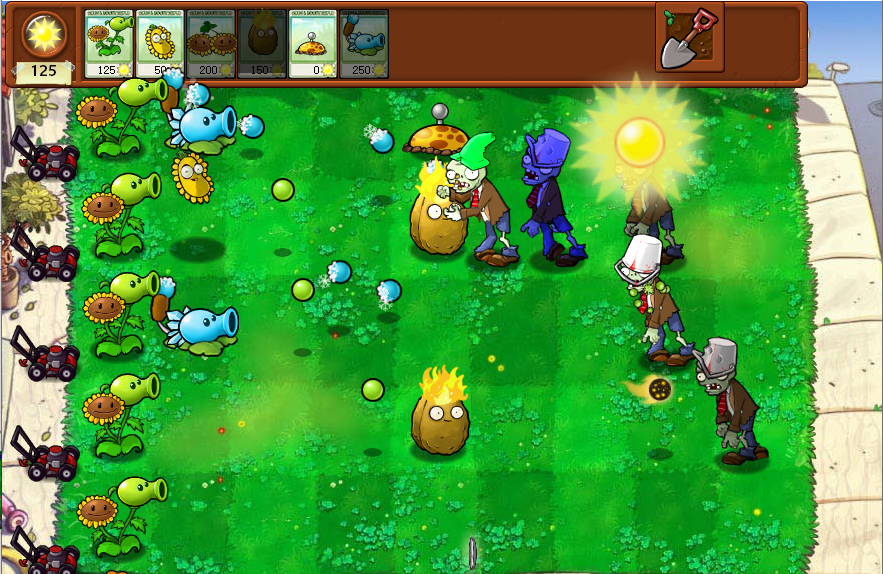


**Competitive Game: Tetris (Multiplayer Version).**

**Game screenshot:**


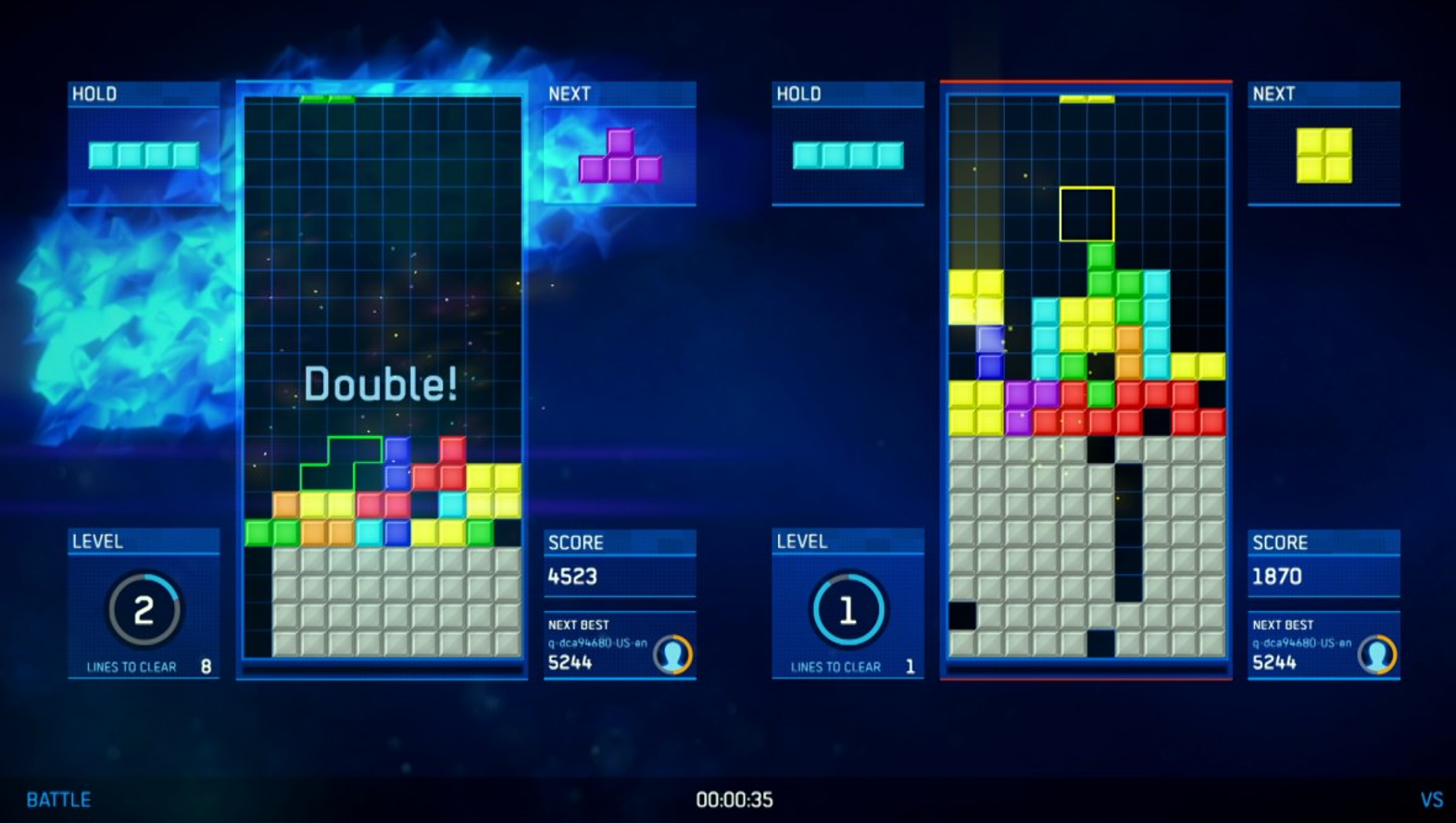

Supplement: Supplementary file 1 [file Supplementary_file_1.docx]
